# LPS-induced expression and release of monocyte tissue factor in patients with haemophilia

**DOI:** 10.1007/s00277-020-04075-6

**Published:** 2020-05-19

**Authors:** Katharina Holstein, Anna Matysiak, Leonora Witt, Bianca Sievers, Lennart Beckmann, Munif Haddad, Thomas Renné, Minna Voigtlaender, Florian Langer

**Affiliations:** 1 Department of Haematology and Oncology, University Cancer Centre Hamburg (UCCH), University Medical Centre Eppendorf, Hamburg, Germany; 2 Institute of Clinical Chemistry and Laboratory Medicine, University Medical Centre Eppendorf, Hamburg, Germany

**Keywords:** Tissue factor, Monocytes, Microvesicles, Haemophilia, Hepatitis

## Abstract

In haemophilia, thrombin generation and fibrin deposition upon vascular injury critically depend on the tissue factor (TF)-driven coagulation pathway. TF expression by monocytes/macrophages and circulating microvesicles contributes to haemostasis, thrombosis and inflammation. Inflammation is a hallmark of blood-induced joint disease. The aim of this study is to correlate TF production by whole-blood monocytes with inflammatory markers and clinical parameters in patients with moderate-to-severe haemophilia A or B (*n* = 43) in comparison to healthy males (*n* = 23). Monocyte TF antigen and microvesicle-associated TF procoagulant activity (MV TF PCA) were measured immediately after blood draw (baseline) and following incubation of whole blood with buffer or lipopolysaccharide (LPS) using two-colour flow cytometry and chromogenic FXa generation assay, respectively. Patients with HIV or uncontrolled HBV/HCV infections were excluded. TF was hardly detectable and not different in baseline and buffer-treaded samples from both groups. Stimulation with LPS, however, induced monocyte TF production, with increased TF-specific mean fluorescence intensity (*P* = 0.08) and MV TF PCA (*P* < 0.05) in patients compared to controls. Patients also had elevated hs-CRP and IL-6 serum levels (*P* < 0.001), which correlated with LPS-induced TF parameters. Further exploratory analyses revealed that the presence of systemic (low-grade) inflammation and boosted LPS-induced monocyte TF production were mainly restricted to patients with clinically controlled HBV and/or HCV infection (*n* = 16), who were older and also had a significantly worse orthopaedic joint score than patients with no history of viral hepatitis (*P* < 0.01). Our study delineates a previously unrecognised link between systemic inflammation and inducible monocyte TF production in patients with haemophilia A or B.

**Electronic supplementary material:**

The online version of this article (10.1007/s00277-020-04075-6) contains supplementary material, which is available to authorized users.

## Introduction

Haemophilia A and B are X-linked recessive bleeding disorders caused by deficiencies in clotting factor VIII (FVIII) or IX (FIX), respectively. Despite initiation of prophylaxis in early childhood, many adolescents and younger adults with moderate-to-severe haemophilia develop irreversible joint damage [1]. Notably, there is tremendous inter-individual variability with regard to the frequency of joint bleeding and the severity of manifesting arthropathy [2]. Moreover, factor trough levels are only poorly correlated with the bleeding rate in patients with severe haemophilia on prophylaxis [3], suggesting that additional, previously unrecognised factors are involved.

Upon injury, coagulation is triggered by the exposure of tissue factor (TF), the cellular receptor and co-factor for FVII/FVIIa [4]. The TF-FVIIa complex not only activates FX, but also FIX and, in complex with FXa, FVIII, thus priming the intrinsic amplification loop of the coagulation protease cascade required for stable fibrin clot formation [4, 5]. While the brain, heart or kidneys show strong TF expression, hardly any TF is found in synovial membranes and skeletal muscles [6], at least partially explaining why patients with haemophilia are particularly prone to recurrent joint and soft-tissue bleeding [7]. The importance of the TF-driven coagulation pathway in haemophilia is underlined by the haemostatic efficacy of monoclonal antibodies targeting TF pathway inhibitor (TFPI) [8].

TF is constitutively expressed at extravascular sites but may be induced on circulating monocytes by inflammatory cytokines or bacterial endotoxins [9]. Activated monocytes also release microvesicles (MVs) that contribute to hypercoagulability [4]. Induction of procoagulant TF-bearing MVs through binding of an Ig-P-selectin fusion protein to monocyte PSGL-1 reverses the bleeding phenotype of mice with severe haemophilia A [10]. TF not only promotes coagulation but also pro-inflammatory cell signalling through protease-activated receptors (PARs) [11].

Intraarticular blood rapidly triggers leukocyte invasion and cytokine production [12–15], resulting in chronic synovitis, which shares both histological and biochemical features with rheumatoid arthritis (RA) [12]. RA is characterized by elevated numbers of TF-positive macrophages in the synovium, and both interstitial fibrin deposition and release of procoagulant MVs into the synovial fluid have been demonstrated [16, 17].

Monocytes and tissue macrophages thus play an important role in maintaining haemostasis in chronically inflamed joint capsules, with cytokines up-regulating TF expression and MV shedding. Of note, similar to mice with severe haemophilia B, wound healing is impaired in low-TF mice [18], further stressing the importance of the TF-driven coagulation pathway not only for haemostasis and thrombosis, but also for inflammation, migration/proliferation and tissue remodelling.

We have previously shown that agonist-induced expression and release of platelet protein disulphide isomerase (PDI), an abundant pro-inflammatory and thrombogenic oxidoreductase involved in TF activation on monocytes/macrophages, are increased in patients with haemophilia A [19], pointing to a potential role of platelet PDI as an injury response signal under conditions of defective thrombin generation. In the present study, we aimed to expand on these findings and used an ex vivo endotoxinaemia model to investigate the expression and release of monocyte TF in patients with moderate-to-severe haemophilia under inflammatory conditions. Because HIV infection and advanced liver disease due to viral hepatitis have been linked to TF-driven coagulation and inflammation [20, 21], patients with known HIV or uncontrolled HBV/HCV infections were excluded.

## Methods

### Patients

The study protocol was approved by the local ethics committee of the city of Hamburg, Germany (no. PV4584). All participants provided written informed consent.

Adult patients with moderate-to-severe haemophilia A or B (FVIII/FIX ≤ 5%) receiving prophylactic or on-demand treatment were eligible. Patients were recruited during their annual follow-up visits routinely scheduled > 48 h after the last factor concentrate infusion. No patient received emicizumab. Patients with acute joint bleeds, as assessed by clinical examination, < 2 weeks or recent surgery < 4 weeks prior to the appointment, known HIV or other symptomatic viral or bacterial infections, or severe renal or hepatic dysfunction were excluded. Controlled hepatitis B (HBV) and C (HCV) infections (i.e. with no clinical evidence for advanced liver cirrhosis, portal hypertension or hepatic coagulopathy and serum levels of bilirubin and liver enzymes < 2-fold the upper limit of normal), however, and presence or history of clotting factor inhibitors were no exclusion criteria. The orthopaedic joint score (OJS) was determined using the physical examination score of the WFH Joint Score [22]. Significant arthropathy was defined by an OJS of > 4. Based on annual bleeding rate and presence of haemophilic arthropathy, patients were categorised as having a mild or severe bleeding phenotype [19]. A severe bleeding phenotype was defined by > 5 treated bleeds during the preceding year and/or clinically significant arthropathy (i.e. OJS > 4). Healthy male controls were recruited from hospital staff.

### Flow cytometry of monocyte TF antigen

Venous blood (3 × 5 mL) was drawn into plastic tubes containing 3.2% trisodium citrate. One tube was immediately analysed (baseline), whereas the other two tubes were incubated with 10 μg/mL *E. coli*-derived lipopolysaccharide (LPS; serotype 0111:B4, Sigma-Aldrich, St. Louis, MO, USA) or phosphate-buffered saline (PBS) for 4 h at 37 °C. The LPS dose of 10 μg/mL was based on a previous report on (pre-) analytical variables affecting the measurement of plasma-derived MV-associated TF activity [23] and on initial studies in our laboratory using isolated peripheral blood mononuclear cells (PBMCs) (Online Resource 1A and B). At a saturating concentration of 10 μg/mL, LPS-induced monocyte TF antigen expression was significantly lower in whole blood as compared to PBMCs (Online Resource 1C). TF antigen on CD14-positive monocytes was analysed by two-colour flow cytometry [24], using the function blocking HTF-1 monoclonal antibody that has previously been shown to specifically recognise TF on LPS-stimulated monocytes [25, 26]. Following subtraction of the non-specific background obtained in the presence of control IgG from the signal received in the presence of TF monoclonal antibody, results were expressed as arbitrary units (AU) of TF-specific mean fluorescence intensity (MFI) or as the proportion (%) of TF-positive cells [representative experiments are shown in Online Resource 2].

### Isolation and analysis of plasma microvesicles

To obtain platelet-poor plasma (PPP), whole-blood was centrifuged for 2 × 10 min at 2060×*g*. PPP was snap-frozen in liquid nitrogen and stored at − 80 °C. Microvesicles (MVs) were isolated from thawed PPP by high-speed centrifugation (2 × 30 min at 16,100×*g*) and analysed for TF-specific procoagulant activity (MV TF PCA) using a chromogenic FXa generation endpoint assay in the presence of 10 nM recombinant FVIIa as previously described [27, 28]. Results were expressed as AU per 200 μL of PPP. Plasma for the analysis of MV TF PCA was not available from one patient and two controls.

### Measurement of hs-CRP and IL-6

High-sensitivity C-reactive protein (hs-CRP) and interleukin-6 (IL-6) were measured in baseline serum samples using the CardioPhase® hs-CRP assay (Siemens Healthcare, Erlangen, Germany) on a Dimension Vista® 1500 system and the Elecsys® IL-6 assay (Roche Diagnostics, Rotkreuz, Switzerland) on a cobas® e 411 analyser, respectively.

### Statistical analysis

Normally and non-normally distributed data were presented as mean ± standard deviation (SD) and median with (inter-quartile) range, respectively, and analysed using the two-sided Student’s *t* test or the Mann-Whitney *U* test. For multiple comparisons, ANOVA and Tukey’s post hoc test or the Kruskal-Wallis and Dunn’s post hoc test were used. Categorical data were analysed using the Fisher’s exact test. Correlation coefficients were according to the methods of Pearson or Spearman. A *P* value of < 0.05 was considered statistically significant. All analyses were performed using GraphPad Prism Software (San Diego, CA, USA) version 7.0.

## Results

### Study cohort

We included 43 patients and 23 healthy males. Clinical patient characteristics are shown in Table 1. The majority of patients (79%) had haemophilia A, and most (91%) had severe clotting factor deficiency. At study inclusion, 35 patients (81%) received prophylactic replacement therapy, while 8 patients (19%) were treated on demand. A history of clotting factor inhibitor was present in 3 patients (7%), and 16 patients (37%) had been tested positive for HBV and/or HCV infection. Twenty-two patients (51%) had significant arthropathy, with target joints and clinical signs of synovitis being present in 3 (7%) and 6 patients (14%), respectively. While whole-blood platelet and monocyte counts were similar between both groups, total leukocyte and granulocyte counts were significantly increased in the patient cohort.

**Table 1:** Clinical patient characteristics

	Patients	Controls	*P* value
*N*	43	23	
Age in years, mean ± SD	33.9 ± 12.8	35.6 ± 11.6	0.61
Blood counts, mean ± SD			
Haemoglobin, g/dL	15.2 ± 0.9	15.4 ± 1.3	0.33
Platelets, 1 × 10^9^/L	240.6 ± 53.8	230.7 ± 60.7	0.50
Leukocytes, 1 × 10^9^/L	6.6 ± 1.8	5.7 ± 1.1	**0.03**
Granulocytes, 1 × 10^9^/L*	4.09 ± 1.37	3.39 ± 0.79	**0.03**
Monocytes, 1 × 10^9^/L*	0.43 ± 0.18	0.45 ± 0.15	0.66
Type of haemophilia, no. (%)			
A	34 (79)		
B	9 (21)		
Severity, no. (%)			
Moderate	4 (9)		
Severe	39 (91)		
Current replacement therapy, no. (%)			
Prophylaxis	35 (81)		
On demand	8 (19)		
History of inhibitor, no. (%)	3 (7)		
Infections, no. (%)			
HIV	0 (0)		
HBV	3 (7)		
HCV	4 (9)		
HBV + HCV	9 (21)		
Total bleeds, median (range)^&^	2 (0–22)		
Joint bleeds, median (range)^&^	2 (0–7)		
Target joints, no. (%)^#^	3 (7)		
Orthopaedic joint score, median (range)^§^	5 (0–39)		
Clinically significant arthropathy, no. (%)^¶^	22 (51)		
Clinical diagnosis of synovitis, no. (%)	6 (14)		
Bleeding phenotype, no. (%)^†^			
Mild	17 (40)		
Severe	26 (60)		

*P* values are according to two-sided Student’s *t* test. Abbreviations are as follows: HBV, hepatitis B virus; HCV, hepatitis C virus; HIV, human immunodeficiency virus; SD, standard deviation

*Granulocyte and monocyte counts were not available for 6 patients

^&^Numbers for total bleeds and joint bleeds refer to the year before study inclusion

^#^Three or more bleeds into the same joint within 6 months

^§^The orthopaedic joint score (OJS) was determined using the physical examination score of the World Federation of Haemophilia (WFH) Joint Score, which assesses elbows, knees and ankles for swelling, muscle atrophy, axial deformity, crepitus on motion, range of motion, flexion contracture and instability. The sum score ranges from 0 to 68, with higher values indicating more severe haemophilic arthropathy [22]

^¶^OJS of > 4

^†^A severe bleeding phenotype was defined by > 5 treated bleeding episodes during the preceding year and/or an OJS of > 4

### Monocyte TF antigen

**Fig. 1:**
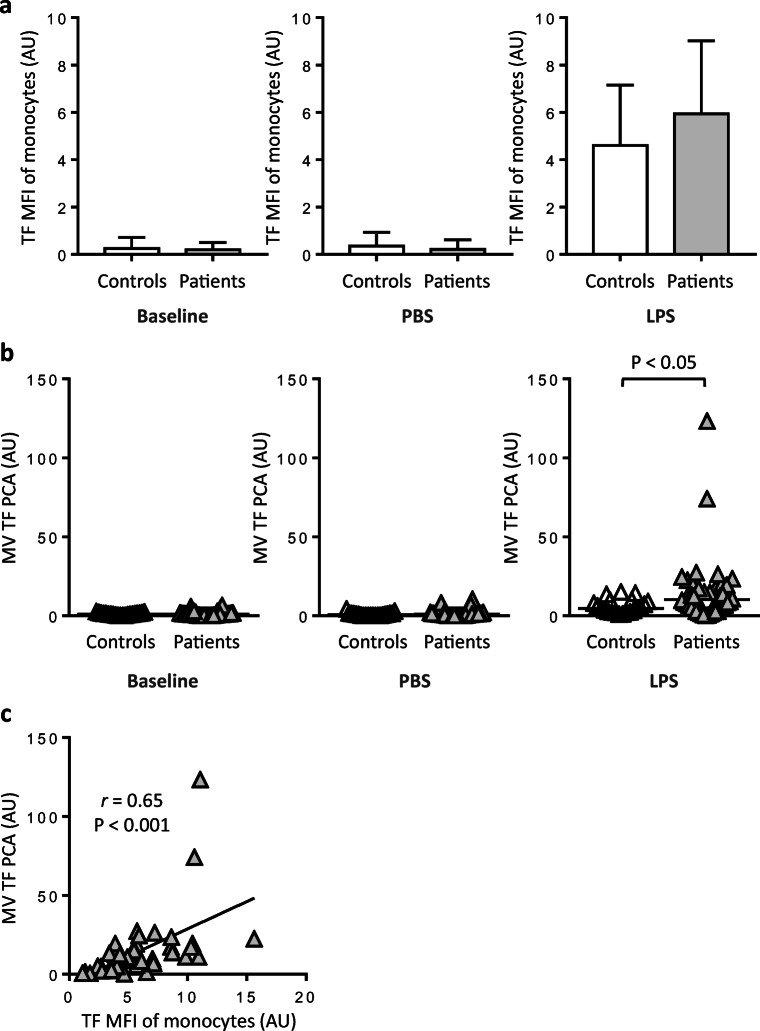
Monocyte TF antigen expression and release of MV-associated TF PCA in patients and controls. **a** TF antigen on whole-blood monocytes was analysed by two-colour flow cytometry both at baseline and after incubation for 4 h at 37 °C with buffer (PBS) or lipopolysaccharide (LPS). AU denotes arbitrary units. **b** Plasma microvesicles (MVs) were isolated from baseline and PBS- or LPS-treated whole blood and analysed for TF-specific procoagulant activity (PCA) using a chromogenic FXa generation endpoint assay. Results are presented as AU per 200 μL of platelet-poor plasma. Horizontal bars indicate median MV TF PCA levels. The *P* value is according to Mann-Whitney *U* test. **c** MV-associated TF PCA was plotted against TF-specific mean fluorescence intensity (MFI) of monocytes. Values were obtained from LPS-treated patient samples. Correlation coefficient (*r*) and *P* value are according to the method of Spearman. Values for MV TF PCA are missing for one patient and two controls

In both patients and controls, virtually no TF antigen was detectable on CD14-positive monocytes in baseline and PBS-treated samples (Fig. 1a and Online Resource 3A). In contrast, stimulation with LPS resulted in robust monocyte TF expression, with mean values (± SD) for TF-specific MFI of 5.9 ± 3.1 AU and 4.6 ± 2.6 AU in patients and controls, respectively (*P* = 0.08) (Fig. 1a). LPS stimulation also increased monocyte TF antigen when reported as percent TF-positive cells (Online Resource 3A). Although not statistically significant, LPS-induced monocyte TF antigen expression was higher in patients compared to controls. As expected, there was a significant correlation between monocyte TF-specific MFI and the proportions of TF-positive monocytes in LPS-treated patient samples (Online Resource 3B).

### MV TF PCA

Similar to monocyte TF antigen, essentially no MV TF PCA was detectable in baseline and PBS-treated samples from both patients and controls (Fig. 1b**)**. Stimulation with LPS, however, increased MV TF PCA, with median levels being significantly higher in patients vs. controls (10.2 vs. 4.6 AU, *P* < 0.05). In LPS-treated patient samples, there was a significant correlation between monocyte TF antigen, expressed as either TF-specific MFI (Fig. 1c) or percent TF-positive cells (Online Resource 3C), and MV TF PCA. Collectively, these findings indicate that stimulation of whole blood with LPS resulted in the enhanced release of monocyte-derived TF-bearing MVs in patients with haemophilia A or B.

### hs-CRP and IL-6

**Fig. 2:**
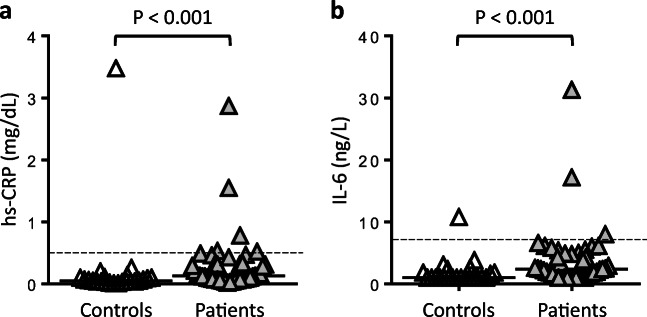
hs-CRP and IL-6 serum levels in patients and controls. Antigen levels of high-sensitivity C-reactive protein (hs-CRP, **a**) and interleukin-6 (IL-6, **b**) were measured in baseline serum samples from patients and controls. Median levels and upper limits of the normal reference ranges are indicated by horizontal bars and dashed lines, respectively. *P* values are according to Mann-Whitney *U* test

Compared to controls, patients had significantly increased serum levels of hs-CRP and IL-6 (Fig. 2). Although most values were still within the respective reference ranges, these findings are consistent with a state of low-grade inflammation in the patient cohort, as also indicated by significantly elevated whole-blood leukocyte and granulocyte counts (Table 1).

### Correlation between LPS-induced TF and inflammatory markers

**Fig. 3:**
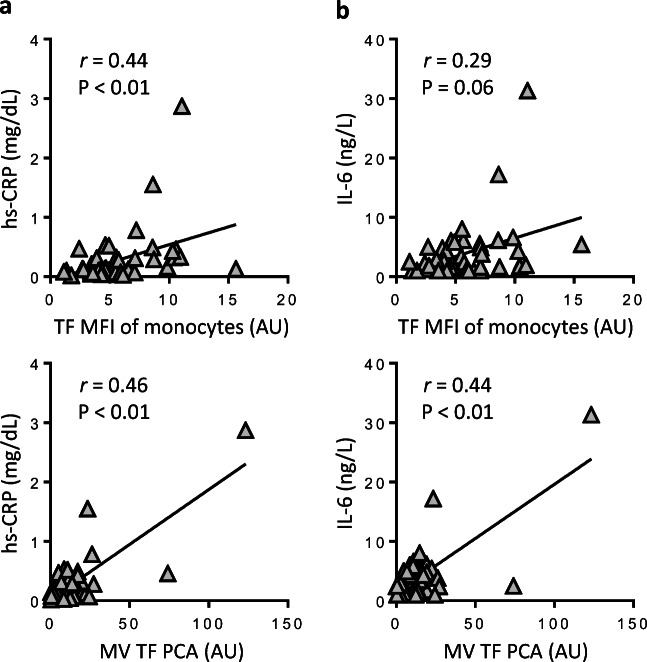
Correlations between hs-CRP/IL-6 and LPS-induced monocyte TF in the patient cohort. Baseline serum levels of hs-CRP (**a**) and IL-6 (**b**) were plotted against monocyte TF antigen, expressed as TF-specific MFI, and release of MV-associated TF PCA. Values were obtained from LPS-treated patient samples. Correlation coefficients (*r*) and *P* values are according to the method of Spearman. AU denotes arbitrary units. The value for MV TF PCA is missing for one patient

Because monocyte TF antigen and MV TF PCA were hardly detectable in baseline and buffer-treated samples (Fig. 1), we correlated inflammatory markers with LPS-induced TF parameters. In the patient cohort, hs-CRP serum levels significantly correlated with both monocyte TF antigen and MV TF PCA (Fig. 3a and Online Resource 4A), whereas for IL-6, only the correlation with MV TF PCA reached statistical significance (Fig. 3b and Online Resource Fig. 4B). In addition, both monocyte TF antigen and MV TF PCA significantly correlated with whole-blood leukocytes in LPS-treated patient samples (Online Resource 5), further supporting a link between LPS-induced monocyte TF production and (low-grade) inflammation in patients with moderate-to-severe haemophilia.

### Association of the HBV/HCV infection status with inflammation and monocyte TF production

Our previous findings indicated that LPS-induced monocyte TF production was associated with low-grade inflammation in patients with moderate-to-severe haemophilia. While HIV-positive patients and those with advanced liver disease due to viral hepatitis were not eligible, the study protocol allowed for the inclusion of patients with clinically controlled HBV and/or HCV infections. In an exploratory post hoc analysis, we therefore asked whether the HBV/HCV infection status played a role in this association.

Sixteen from 43 patients (37%) had positive HBV and/or HCV test results at study inclusion. All of these patients had bilirubin and AST serum levels within the respective reference ranges. ALT serum levels were normal in 13 and only slightly elevated in 3 patients.

While 3 and 4 patients had only been tested positive for either HBV or HCV, respectively, the remaining 9 patients had concomitant HBV and HCV infection (Table 1). Of the 12 HBV-positive patients, only one patient, who did not have HCV co-infection, presented with chronic hepatitis with low-grade HBs antigen production, but normal liver function tests, while HBV infection had resolved with protective immunity in the other 11 patients. Of the 13 HCV-positive patients, 7 patients had received successful treatment with absence of HCV viraemia at study inclusion. In 5 treatment-naïve patients, PCR test results were negative despite presence of anti-HCV antibodies, a finding consistent with spontaneous clearance of the virus. The remaining patient had chronic HCV infection with significant viraemia, but normal liver function tests.

Taken together, these findings are consistent with either resolved or clinically controlled HBV/HCV infections in our patient cohort.

**Fig. 4:**
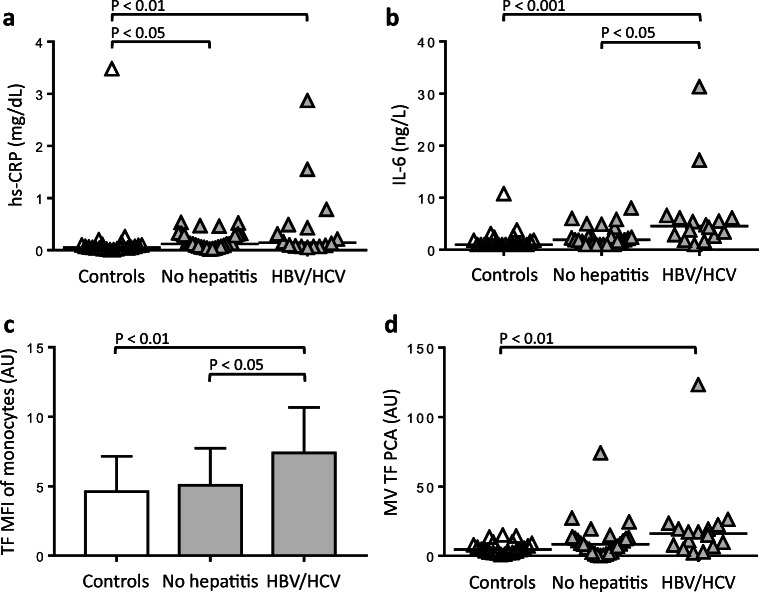
Association of the HBV/HCV infection status with inflammatory markers and LPS-induced monocyte TF. Baseline serum levels of hs-CRP (**a**) and IL-6 (**b**), results for LPS-induced monocyte TF antigen, expressed as TF-specific MFI (**c**), and release of MV-associated TF PCA (**d**) are shown for healthy male controls (*n* = 23) and patients with (*n* = 16) or without positive HBV/HCV test results (*n* = 27) at study inclusion. *P* values are according to ANOVA and Tukey’s post hoc test (**c**) or the Kruskal-Wallis and Dunn’s post hoc test (**a**, **b**, **d**). AU denotes arbitrary units. Values for MV TF PCA are missing for one patient and two controls

While hs-CRP serum levels (Fig. 4a) and whole-blood leukocytes (Online Resource 6A) did not differ between patients with and those without a history of viral hepatitis, HBV/HCV-positive patients (*n* = 16) had significantly higher IL-6 serum levels than HBV/HCV-negative patients (*n* = 27) (Fig. 4b). HBV/HCV-positive patients significantly differed from healthy males with regard to both inflammatory markers and whole-blood leukocytes, while patients with no history of viral hepatitis only had higher hs-CRP serum levels than controls.

When compared to controls, stimulation with LPS resulted in significantly increased monocyte TF antigen (Fig. 4c and Online Resource 6B) and MV TF PCA (Fig. 4d) in HBV/HCV-positive patients. For HBV/HCV-negative patients, none of the TF readouts reached statistical significance. HBV/HCV-positive patients had significantly higher levels of LPS-induced monocyte TF-specific MFI than HBV/HCV-negative patients (Fig. 4c).

HBV/HCV-positive patients were significantly older than HBV/HCV-negative patients (Online Resource 6C). There was no difference in mean age between healthy male controls and HBV/HCV-positive patients, while HBV/HCV-negative patients were significantly younger than controls. Thus, differences in inflammatory markers and LPS-induced monocyte TF production between HBV/HCV-positive patients and healthy male controls cannot be explained by a different age distribution. In line with this conclusion, IL-6 was the only parameter that significantly correlated with age in the total study population (*r* = 0.30, *P* < 0.05; *n* = 66), while no such correlation was found for hs-CRP, whole-blood leukocytes and LPS-induced TF parameters. However, when HBV/HCV-positive patients were excluded from the analysis, there was no correlation between age and IL-6 serum levels (*r* = 0.03, *P* = 0.85; *n* = 50), indicating that IL-6 serum levels per se were not influenced by age in our study cohort.

Taken together, these findings indicate that the presence of low-grade inflammation and increased LPS-induced monocyte TF production in the patient cohort was associated with a positive HBV/HCV infection status.

### Association of haemophilic arthropathy with inflammation, monocyte TF production, and the HBV/HCV infection status

**Fig. 5:**
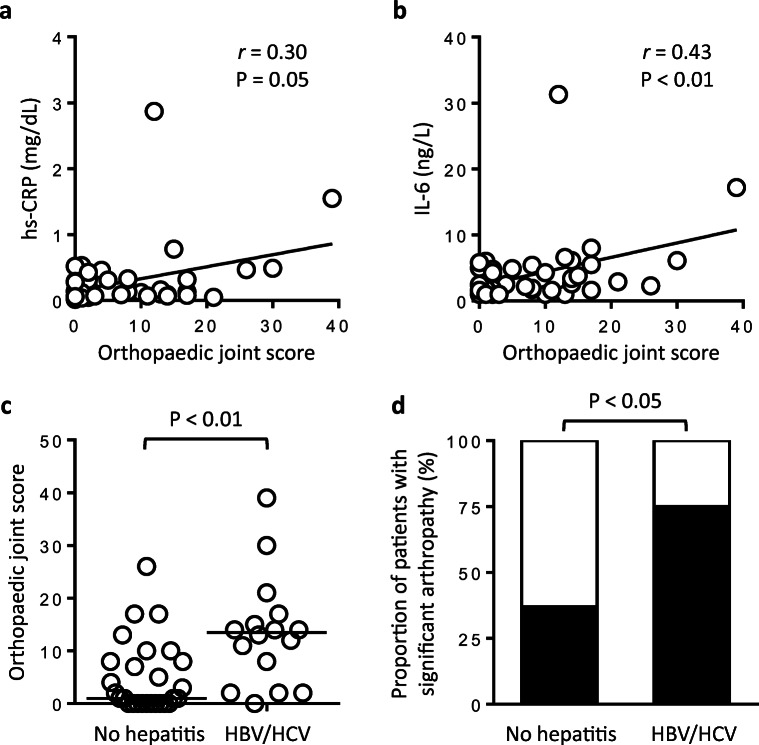
Correlation of the OJS with inflammatory markers and association of haemophilic arthropathy with the HBV/HCV infection status. Baseline serum levels of hs-CRP (**a**) and IL-6 (**b**) were plotted against the orthopaedic joint score (OJS). Correlation coefficients (*r*) and *P* values are according to the method of Spearman. **c** The OJS was assessed in patients with (*n* = 16) or without positive HBV/HCV test results (*n* = 27) at study inclusion. Horizontal bars indicate median OJS. The *P* value is according to Mann-Whitney *U* test. **d** Black bars indicate proportions of patients with clinically significant haemophilic arthropathy, defined by an OJS > 4, as a function of the HBV/HCV infection status. The *P* value is according to Fisher’s exact test

In the patient cohort, the orthopaedic joint score (OJS) correlated with hs-CRP (Fig. 5a) and IL-6 serum levels (Fig. 5b) as well as whole-blood leukocytes (Online Resource 7A). Patients with clinically significant arthropathy (*n* = 22), as defined by an OJS > 4, were older (Online Resource 7B) and had higher IL-6 serum levels (3.6 (1.9–6.1) vs. 1.8 (1.0–3.4) ng/L, median (IQR); *P* < 0.05) than patients without clinically significant arthropathy (*n* = 21). There was a strong trend towards increased LPS-induced monocyte TF-specific MFI in patients with clinically significant arthropathy (6.8 ± 3.4 vs. 5.0 ± 2.5 AU; *P* = 0.05), while no differences were observed with regard to hs-CRP serum levels, whole-blood leukocytes and MV TF PCA. HBV/HCV-positive patients had a significantly worse OJS than HBV/HCV-negative patients (Fig. 5c). Accordingly, the proportion of patients with clinically significant arthropathy was higher in patients with than in those without a positive HBV/HCV infection status (Fig. 5d).

These findings indicate that advanced joint disease and a positive HBV/HCV infection status are closely interrelated and suggest that both conditions contribute to low-grade inflammation and boosted LPS-induced TF production in our patient cohort.

### Association of LPS-induced monocyte TF production with the bleeding phenotype

Patients with a severe bleeding phenotype (*n* = 26) were older (37.7 ± 12.6 vs. 28.1 ± 11.1 years; *P* < 0.05) and had higher IL-6 serum levels (3.2 (1.7–5.7) vs. 1.8 (1.0–3.4) ng/L; *P* < 0.05) than patients with a mild bleeding phenotype (*n* = 17). No differences were observed with regard to hs-CRP serum levels, whole-blood leukocytes and any of the LPS-induced TF parameters. Albeit not significant, the proportion of HBV/HCV-positive patients was higher in patients with a severe than in those with a mild bleeding phenotype (42.3 vs. 29.4%, *P* = 0.52). In addition, no differences with regard to LPS-induced TF parameters, inflammatory markers and OJS were observed between patients receiving prophylactic (*n* = 35) or on-demand (*n* = 8) clotting factor replacement therapy at the time of study inclusion.

## Discussion

In this study, we investigated LPS-induced monocyte TF production in 43 patients with moderate-to-severe haemophilia and found that expression of TF antigen and release of MV-associated TF PCA correlated with markers of systemic (low-grade) inflammation.

Our study was based on the original hypothesis that patients with increased LPS-induced monocyte TF production had a milder bleeding phenotype than patients with a less pronounced response, which we could not confirm. It must be considered, however, that there is no consistent definition of the bleeding phenotype [29], and our definition might be oversimplified, not taking into account that patients on effective prophylaxis hardly bleed and usually have no significant joint disease. We also used peripheral blood monocytes as a surrogate for tissue macrophages, which are key components of the haemostatic envelope. While (monocyte-derived) plasma MVs promote thrombosis in various disease states [9], their contribution to haemostasis, at least under normal, non-inflammatory conditions, is less clear. On the one hand, Hoffman et al. [30] found accumulation of blood-borne TF in a mouse model of venous thrombosis, but not within the haemostatic plugs formed after skin punch biopsy. On the other hand, continuously circulating TF likely plays a role in low-level stimulation of the coagulation protease cascade with “idling” of the clotting system, which may allow for a rapid haemostatic response upon vascular injury [31–33].

Although TF production by monocytes in response to inflammatory stimuli is highly variable [34, 35], an individual remains a high or a low responder for several years [36, 37], suggesting a low degree of intra-individual variability and a possible (poly-) genetic predisposition. In our study, LPS-induced monocyte TF antigen was normally distributed in both patients and controls, and we did not find a clear distinction between high and low responders. Furthermore, because no serial measurements have been performed, we cannot comment on the intra-individual variability of obtained findings. Since TF expression by whole-blood monocytes is a tightly regulated process involving platelets, granulocytes and erythrocytes [38–41], subtle variations in experimental procedures may account for seemingly discrepant findings between independent studies.

For the first time, we delineate an association of LPS-induced monocyte TF production with inflammatory markers in patients with moderate-to-severe haemophilia. From this observation, the question arises of whether systemic inflammation primes peripheral blood monocytes, leading to boosted TF synthesis in response to LPS, or whether monocyte TF itself is directly involved in the elaboration of an inflammatory state. A recent study has placed a subset of TF-expressing monocytes in the epicentre of inflammation and coagulation in chronic HIV infection, with PARs being a potential link between TF-driven coagulation and inflammation [20].

While patients with HIV infection were not eligible for participation in the study, findings of our exploratory post hoc analysis indicate that low-grade inflammation and boosted LPS-induced monocyte TF production were mainly restricted to HBV/HCV-positive patients (Fig. 4). Increased TF expression has been implicated in the pathogenesis of inflammation and fibrosis in advanced HCV infection [21]. Hodowanec et al. [42] found that circulating microparticle-associated TF activity (MP TF) was more frequently detectable in patients with chronic HCV infection than in patients with HIV mono-infection or HIV/HCV co-infection with cleared HCV. Interestingly, MP TF was also associated with advanced liver fibrosis and cellular markers of immune activation. Since no healthy controls were included in the study by Hodowanec et al., it remains speculative whether MP TF is more frequently detectable in patients with cleared HCV than in healthy controls. In our study, only one HCV-positive patient had significant viraemia. Using flow cytometry, a different study found that patients with HCV-related liver cirrhosis and portal vein thrombosis (PVT) had significantly increased monocyte TF antigen expression than cirrhosis patients without PVT or healthy controls [43]. Patients with HCV-related liver cirrhosis were not included in our study.

**Fig. 6:**
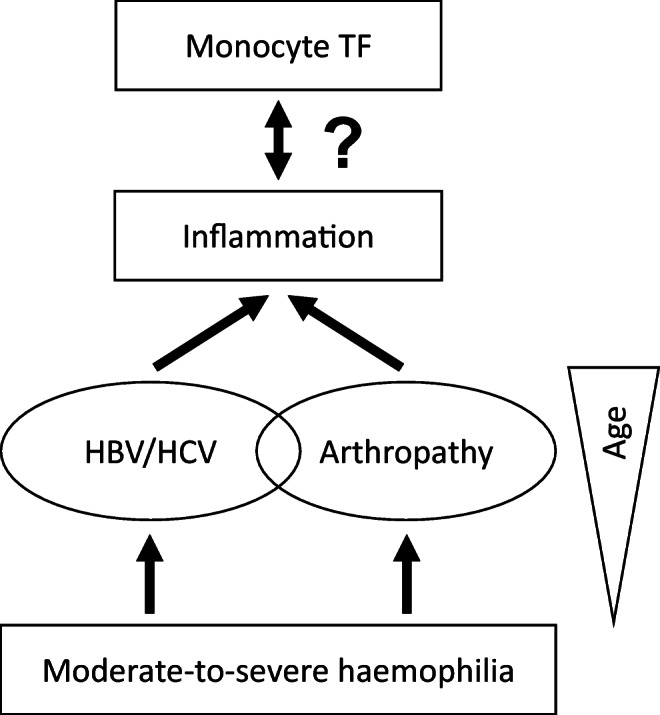
Hypothetical role of monocyte TF in haemophilia-associated inflammation. In adult patients with moderate-to-severe haemophilia, clinically significant arthropathy and a positive HBV/HCV infection status are still frequently encountered complications caused by the bleeding disorder and its treatment, respectively. Both conditions contribute to systemic (low-grade) inflammation and share advanced patient age as a common determinant. Based on our current study and previous literature on the role of monocyte/macrophage TF in thrombo-inflammation, we hypothesise that pro-inflammatory pathways prime whole-blood monocytes to boost TF production in response to pathophysiological stimuli (a desirable effect in the context of deficient thrombin generation and fibrin formation), while monocyte TF may reciprocally fuel inflammation, e.g. through PAR-dependent signalling, thus potentially contributing to synovitis and hepatitis

Taken together, the existing literature on the role of (monocyte) TF in the pathogenesis of coagulation activation and inflammation in non-haemophilic patients with HCV infection provides a basis for our observations. We obtained similar findings when the 3 patients with HBV mono-infection were excluded [not shown]. Considering that HCV was cleared in all but one patient with a positive HCV test result, the cellular and molecular pathways driving low-grade inflammation and boosted LPS-induced monocyte TF production in these patients remain obscure. However, since hs-CRP and IL-6 serum levels also correlated with the orthopaedic joint score (OJS), it is highly likely that the pathophysiology of inflammation was multifactorial in our patient cohort, with blood-induced joint disease playing an additional role [12–15]. Interestingly, a recent study has demonstrated that the presence of HCV infection positively correlated with radiological evidence of arthropathy in 146 Taiwanese patients with moderate-to-severe haemophilia [44], further supporting our hypothesis that a history of viral hepatitis and clinically relevant arthropathy, both of which are more frequently observed in the ageing haemophilia population, closely cooperate in the generation of an inflammatory state (Fig. 6).

In line with this hypothesis, an exploratory analysis of covariance adjusting for patient age and OJS revealed that the HBV/HCV infection status was not independently associated with low-grade inflammation and LPS-induced monocyte TF production in our patient cohort [not shown]. The validity of this analysis, however, is limited by rather small sample sizes and other potential confounders affecting (TF-driven) systemic inflammation and joint health in patients with moderate-to-severe haemophilia (e.g. body weight, blood pressure, smoking and diet habits, level of physical activity). In either case, findings obtained after exclusion of HBV/HCV-positive patients and further restricting our analysis to patients with severe haemophilia A and no clinical evidence for significant arthropathy do not support the concept that inherited FVIII deficiency per se is associated with boosted LPS-induced monocyte TF production (Online Resource 8), which rather results from an inflammatory environment triggered, for instance, by blood-induced joint disease and/or HBV/HCV infections (Fig. 6).

Our study has several limitations. First, despite prospectively defined exclusion criteria the patient population is highly heterogeneous. While heterogeneity of study participants entails a significant risk of confounding, it may actually increase the generalisability of our findings. Second, instead of using imaging tools such as ultrasonography, acute joint bleeds were excluded by clinical judgement only and may thus have occasionally been missed at study inclusion. Third, our arbitrary definition of the bleeding phenotype is rather simple and certainly does not encompass the full complexity of haemorrhage in haemophilia. Fourth, because only adults were included, we cannot comment on LPS-induced monocyte TF production in children and adolescents, who, at least in developed countries, usually do not suffer from advanced arthropathy or blood-borne infections. Finally, although we consider the risk of bias to be low, the laboratory personnel responsible for TF analysis was not blinded with regard to patient and control samples, but was unaware of any other laboratory, demographic and clinical patient characteristics.

In summary, we provide novel evidence that patients with moderate-to-severe haemophilia show boosted TF expression and release by whole-blood monocytes in response to LPS, a finding predominantly observed in patients with a positive HBV/HCV infection status and clinically significant arthropathy and consistent with our previous observation that ADP-induced platelet PDI expression positively correlated with age in patients with haemophilia A [19]. Our findings may thus stimulate further research into the link between systemic inflammation and inducible monocyte TF production, which could be of pathophysiological relevance in the development and progression of blood-induced joint disease.

## Electronic supplementary material

ESM 1 (PDF 102 kb)

ESM 2 (PDF 861 kb)

## Data Availability

Additional data available as electronic supplementary material.
