# Supplementary material for: LPS-induced expression and release of monocyte tissue factor in patients with haemophilia
Source: Ann Hematol. 2020 May 19;99(7):1531–42. doi: 10.1007/s00277-020-04075-6 (PMC7316670; doi:10.1007/s00277-020-04075-6)
Supplement: Supplementary file 1 — (PDF 102 kb) [file 277_2020_4075_MOESM1_ESM.pdf]

LPS-induced expression and release of monocyte tissue factor in patients with haemophilia

Annals of Hematology

Katharina Holstein, Anna Matysiak, Leonora Witt, Bianca Sievers, Lennart Beckmann, Munif Haddad, Thomas Renné, Minna Voigtlaender, Florian Langer

Corresponding author:

Florian Langer MD
Department of Haematology and Oncology
University Cancer Centre Hamburg (UCCH)
University Medical Hamburg-Eppendorf
D-20246 Hamburg, Germany
E-mail: langer@uke.de

Legends to electronic supplementary material

Online Resource 1: LPS dose titration and comparison of whole-blood monocytes to PBMCs

(**A** and **B**) Peripheral blood mononuclear cells (PBMCs) were isolated from citrate-anticoagulated whole blood and stimulated with various concentrations of lipopolysaccharide (0–10 µg/mL) for 4 h at 37°C. Expression of TF antigen on CD14-positive monocytes was subsequently analysed by two-colour flow cytometry. Results are presented as TF-specific mean fluorescence intensity (MFI, **A**) or as the proportion of TF-positive cells (**B**). A representative experiment with different scaling of the x-axis is shown. (**C**) Citrate-anticoagulated whole blood or isolated PBMCs were incubated with buffer (PBS) or 10 µg/mL LPS for 4 h at 37°C before monocyte TF antigen was analysed by flow cytometry (mean ± SD, n=10). Results are presented as TF-specific MFI (left panel) or percent TF-positive cells (right panel). P values are according to two-sided Student's *t*-test.

Online Resource 2: Analysis of monocyte TF antigen by two-colour flow cytometry

(**A**) The region R1 indicates whole-blood monocytes labelled with a fluorescein isothiocyanate (FITC)-conjugated monoclonal antibody against CD14. (**B**) Gated monocytes were stained for TF antigen using a phycoerythrin (PE)-conjugated monoclonal antibody (anti-TF) in comparison to PE-conjugated control IgG. Samples were analysed immediately after blood draw (baseline) and after incubation for 4 h with buffer (PBS) or 10 µg/mL lipopolysaccharide (LPS). TF-positive monocytes were identified by a gate arbitrarily set to include 1 % of monocytes treated with control IgG. Representative histograms from a healthy male control and a patient with haemophilia A are shown. Arbitrary units (AU) of TF-specific mean fluorescence intensity (MFI) and percentages of TF-positive monocytes, obtained after subtracting values in the presence of control IgG from those in the presence of anti-TF, are indicated in red.

Online Resource 3: Monocyte TF antigen expression in patients and controls and correlation of TF parameters in LPS-treated patient samples

(**A**) TF antigen on whole-blood monocytes was analysed by two-colour flow cytometry both at baseline and after incubation for 4 h at 37°C with buffer (PBS) or lipopolysaccharide (LPS). Results are presented as percent TF-positive monocytes. (**B**) The proportion of TF-positive monocytes was plotted against TF-specific MFI of monocytes in LPS-treated patient samples. Correlation coefficient (*r*) and P value are according to the method of Pearson. (**C**) MV-associated TF PCA was plotted against TF-positive monocytes in LPS-treated patient samples. Correlation coefficient (*r*) and P value are according to the method of Spearman. The value for MV TF PCA is missing for one patient.

Online Resource 4: Correlations between hs-CRP/IL-6 and LPS-induced monocyte TF antigen in the patient cohort

Baseline serum levels of hs-CRP (**A**) and IL-6 (**B**) were plotted against monocyte TF antigen,

expressed as the proportion of TF-positive cells. Values were obtained from LPS-treated patient samples. Correlation coefficients (*r*) and P values are according to the method of Spearman.

Online Resource 5: Correlation between whole-blood leukocytes and LPS-induced TF parameters in the patient cohort

Baseline whole-blood leukocytes were plotted against monocyte TF antigen, expressed as TF-specific MFI (**A**) or percent TF-positive cells (**B**), and MV-associated TF PCA (**C**). Values were obtained from LPS-treated patient samples. Correlation coefficients (*r*) and P values are according to the method of Pearson (**A** and **B**) or Spearman (**C**). The values for MV TF PCA is missing for one patient.

Online Resource 6: Effect of the HBV/HCV infection status on whole-blood leukocytes, LPS-induced monocyte TF antigen and age in the patient cohort

Baseline whole-blood leukocytes (**A**), LPS-induced monocyte TF antigen, expressed as the proportion of TF-positive cells (**B**), and age (**C**) are shown for healthy male controls (n=23) and patients with (n=16) or without positive HBV/HCV test results (n=27) at study inclusion. P values are according to ANOVA and Tukey´s post-hoc test.

Online Resource 7: Correlation of the OJS with whole-blood leukocytes and effect of clinically significant arthropathy on age in the patient cohort

(**A**) Baseline whole-blood leukocytes were plotted against the orthopaedic joint score (OJS). Correlation coefficient (*r*) and P value are according to the method of Spearman. (**B**) Age is shown for healthy male controls (n=23) and patients with (n=22) or without clinically significant arthropathy (n=21) at study inclusion. P values are according to ANOVA and Tukey´s post-hoc test.

Online Resource 8: Exploratory subgroup analyses of LPS-induced TF parameters and inflammatory markers

Data are presented as mean ± standard deviation or as median and inter-quartile range. *P values (for the comparison of respective patient subgroups with controls) are according to two-sided Student's *t*-test or Mann-Whitney U test. OJS ≤ 4 indicates absence of clinically significant arthropathy. Values for MV TF PCA are missing for one patient and two controls. Abbreviations are as follows (in alphabetical order): AU, arbitrary units; HA, haemophilia A; HB, haemophilia B; HBV, hepatitis B virus; HCV, hepatitis C virus; hs-CRP, high-sensitivity C-reactive protein; IL-6, interleukin-6; LPS, lipopolysaccharide; MFI, mean fluorescence intensity; MV, microvesicle; n.a., not applicable; n.d., not determined; OJS, orthopaedic joint score; PCA, procoagulant activity; TF, tissue factor; w/o, without.
