# Supplementary material for: LPS-induced expression and release of monocyte tissue factor in patients with haemophilia
Source: Ann Hematol. 2020 May 19;99(7):1531–42. doi: 10.1007/s00277-020-04075-6 (PMC7316670; doi:10.1007/s00277-020-04075-6)
Supplement: Supplementary file 2 — (PDF 861 kb) [file 277_2020_4075_MOESM2_ESM.pdf]

LPS-induced expression and release of monocyte tissue factor in patients with haemophilia

Annals of Hematology

Katharina Holstein, Anna Matysiak, Leonora Witt, Bianca Sievers, Lennart Beckmann, Munif Haddad, Thomas Renné, Minna Voigtlaender, Florian Langer

Corresponding author:
Florian Langer MD
Department of Haematology and Oncology
University Cancer Centre Hamburg (UCCH)
University Medical Hamburg-Eppendorf
D-20246 Hamburg, Germany
E-mail: langer@uke.de

Electronic Supplementary Material

Online Resource 1

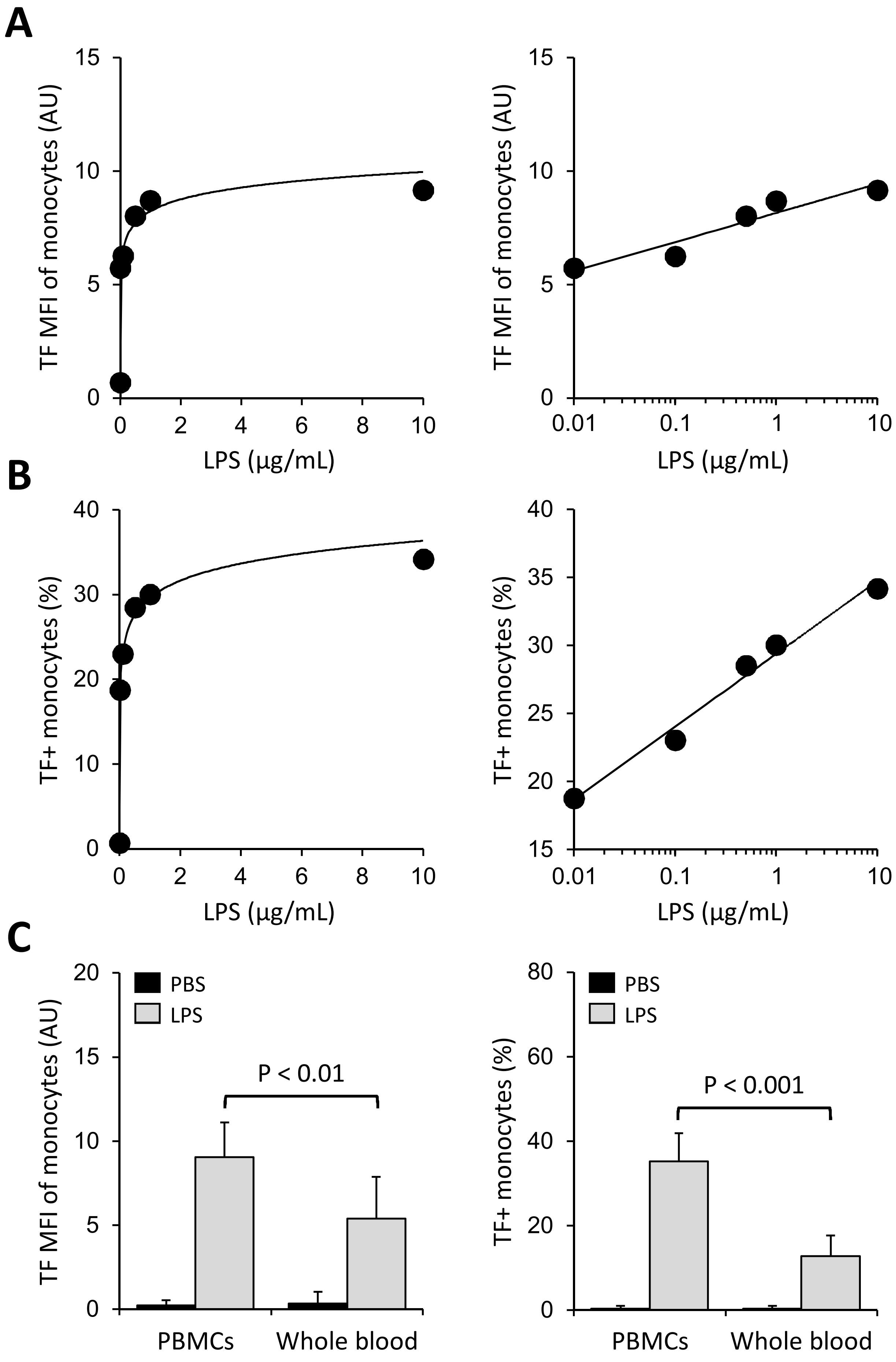

Online Resource 2

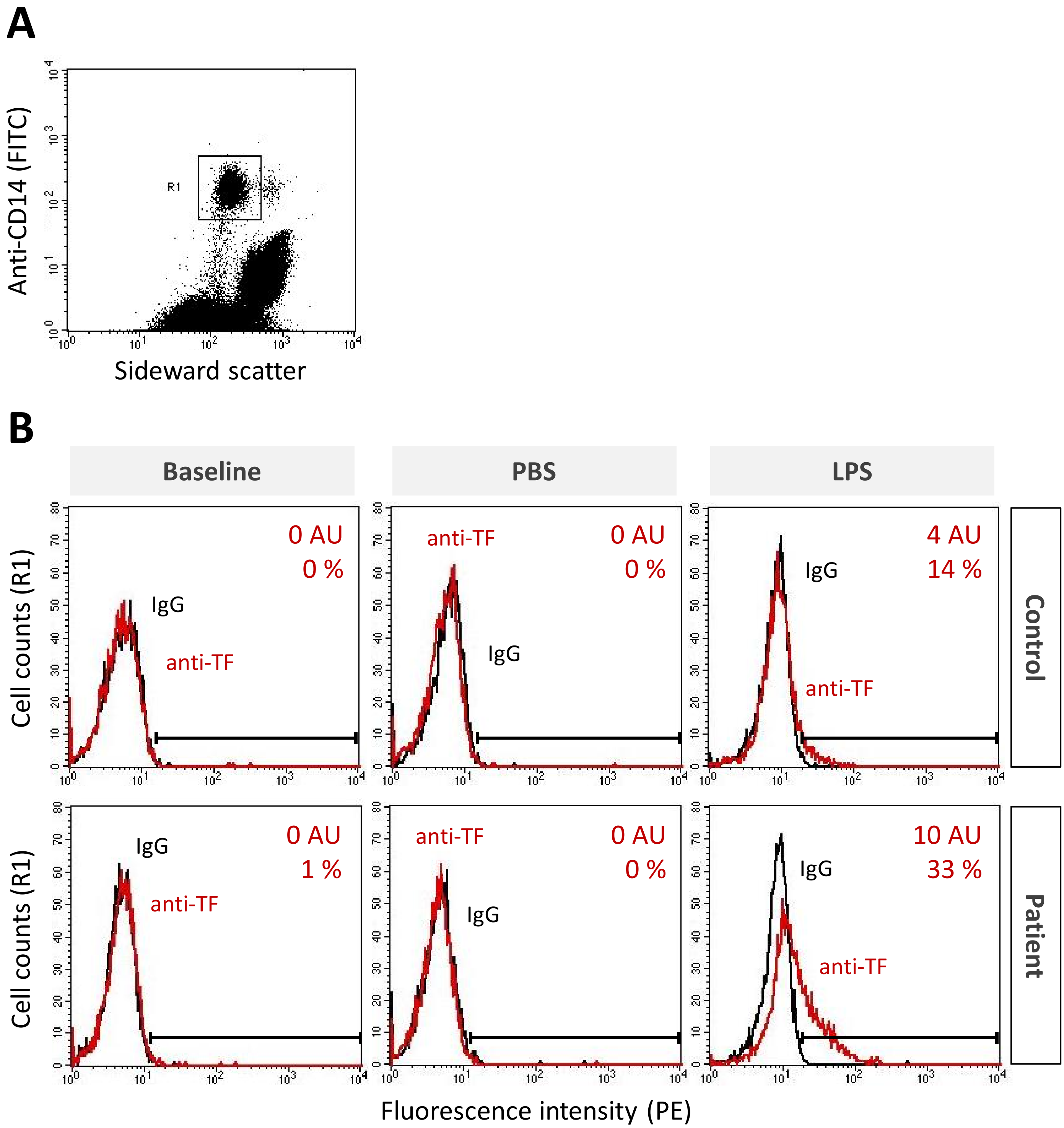

Online Resource 3

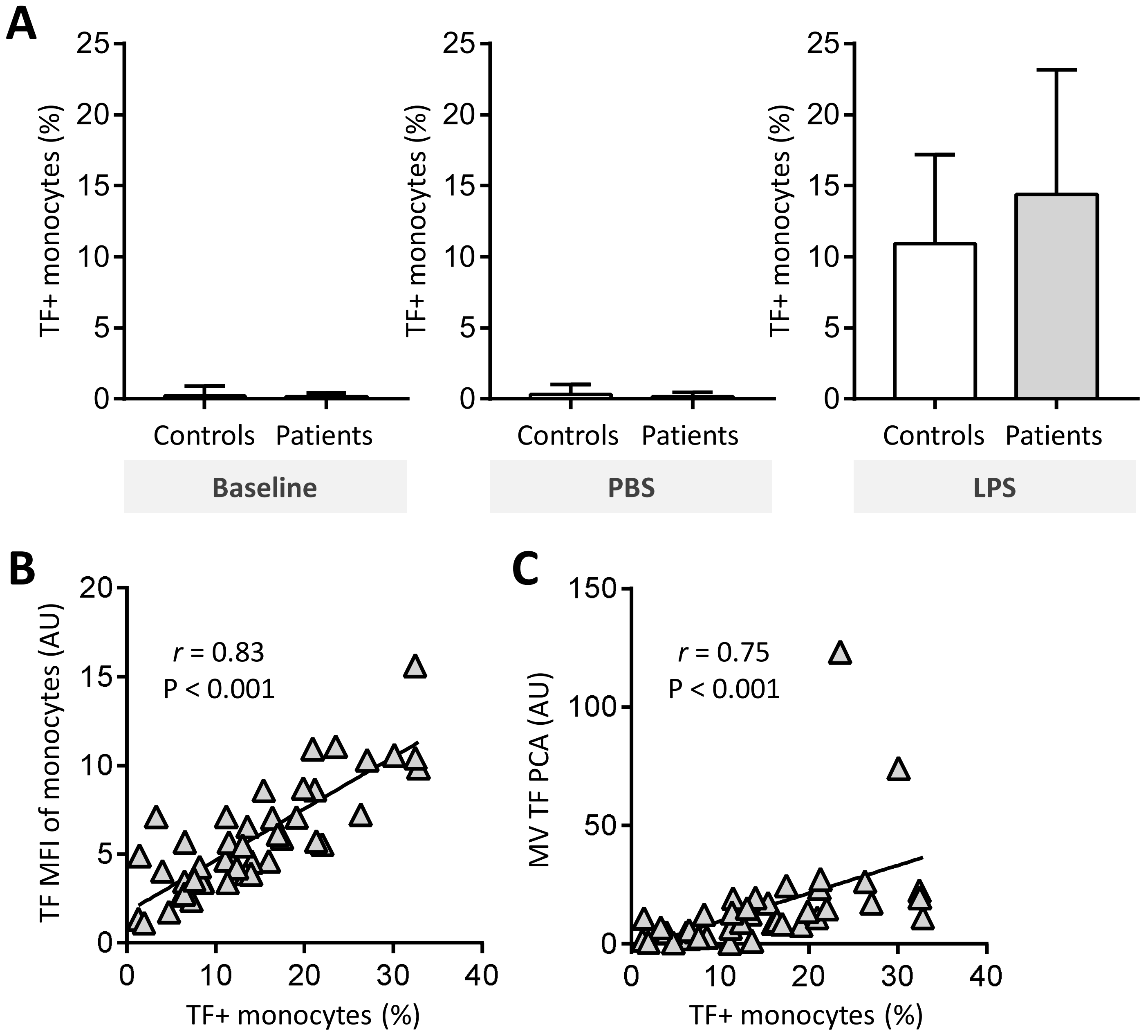

Online Resource 4

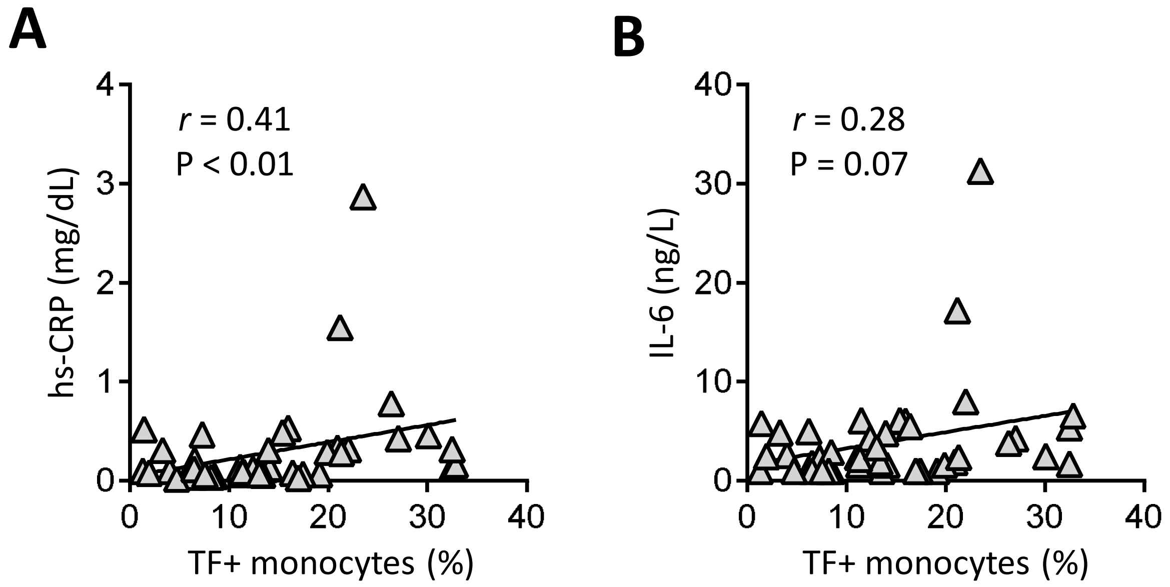

Online Resource 5

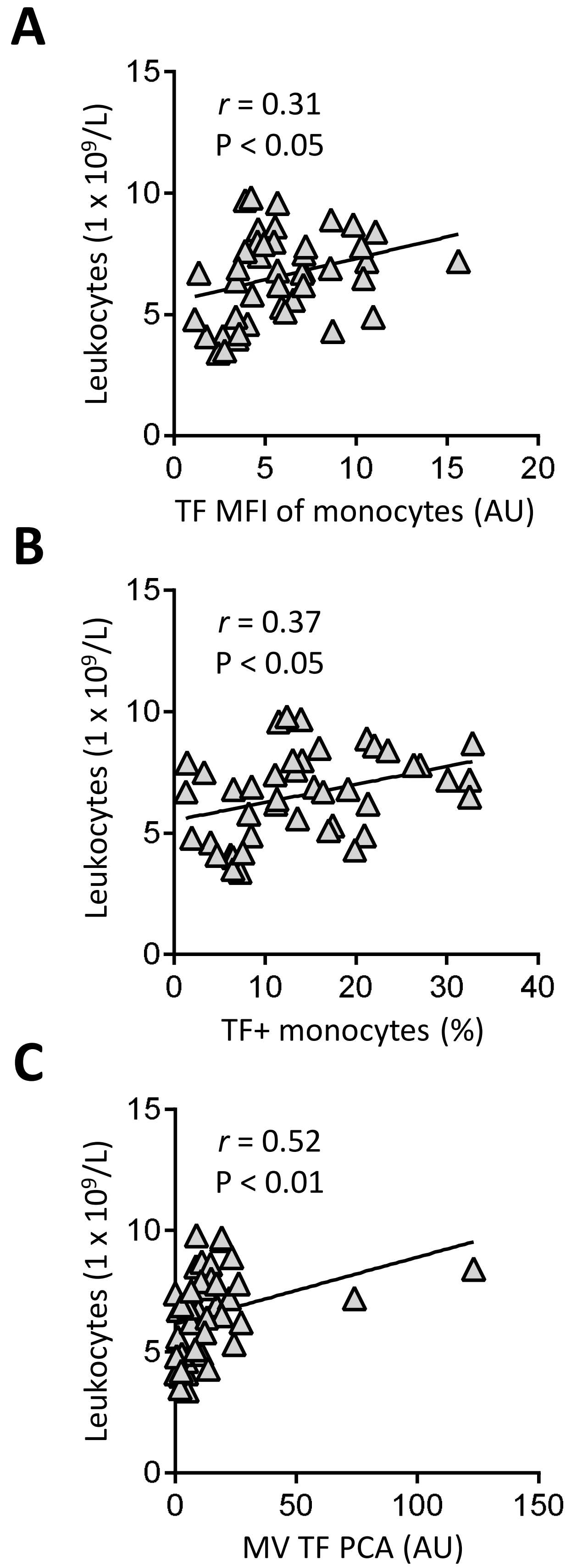

Online Resource 6

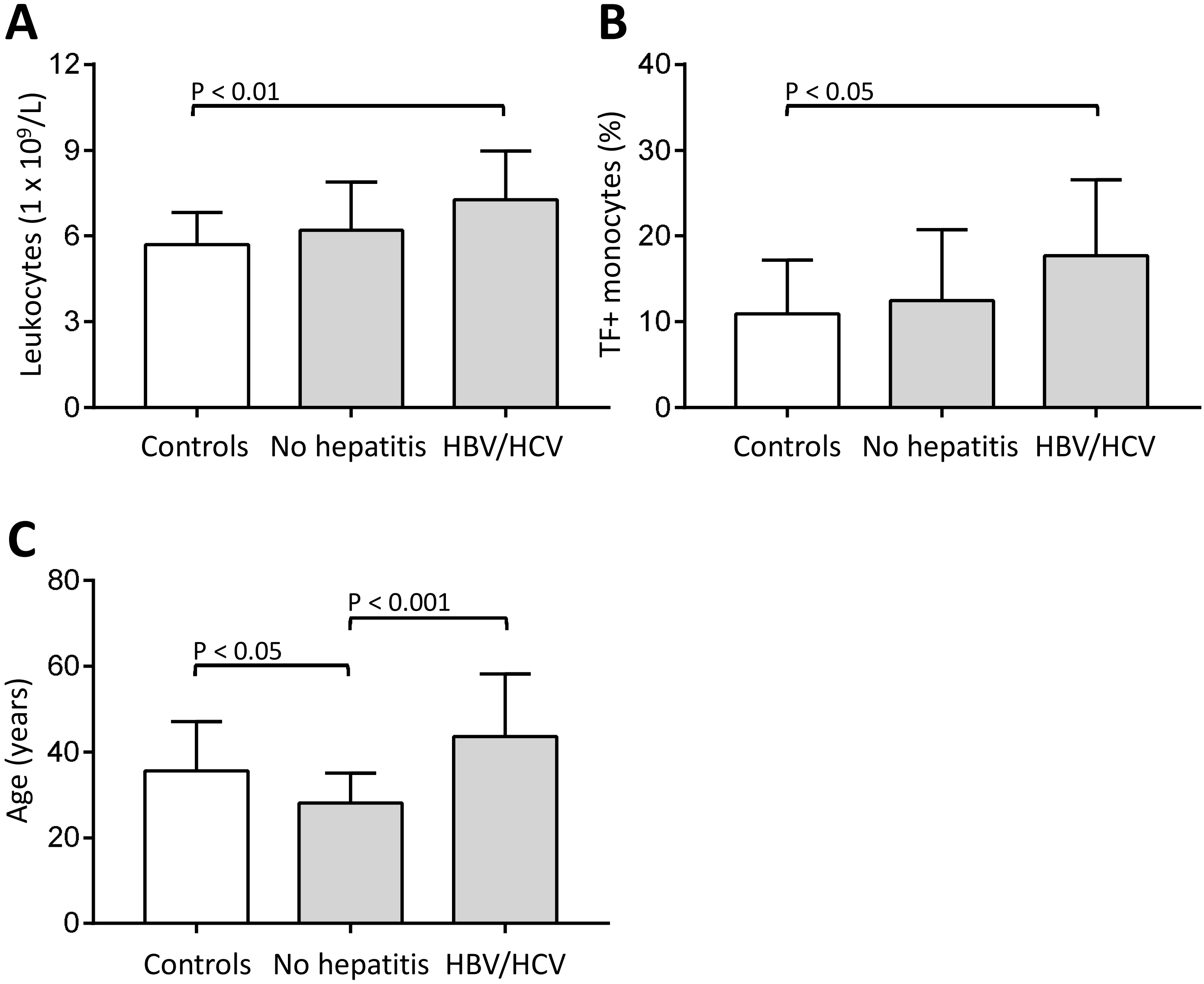

Online Resource 7

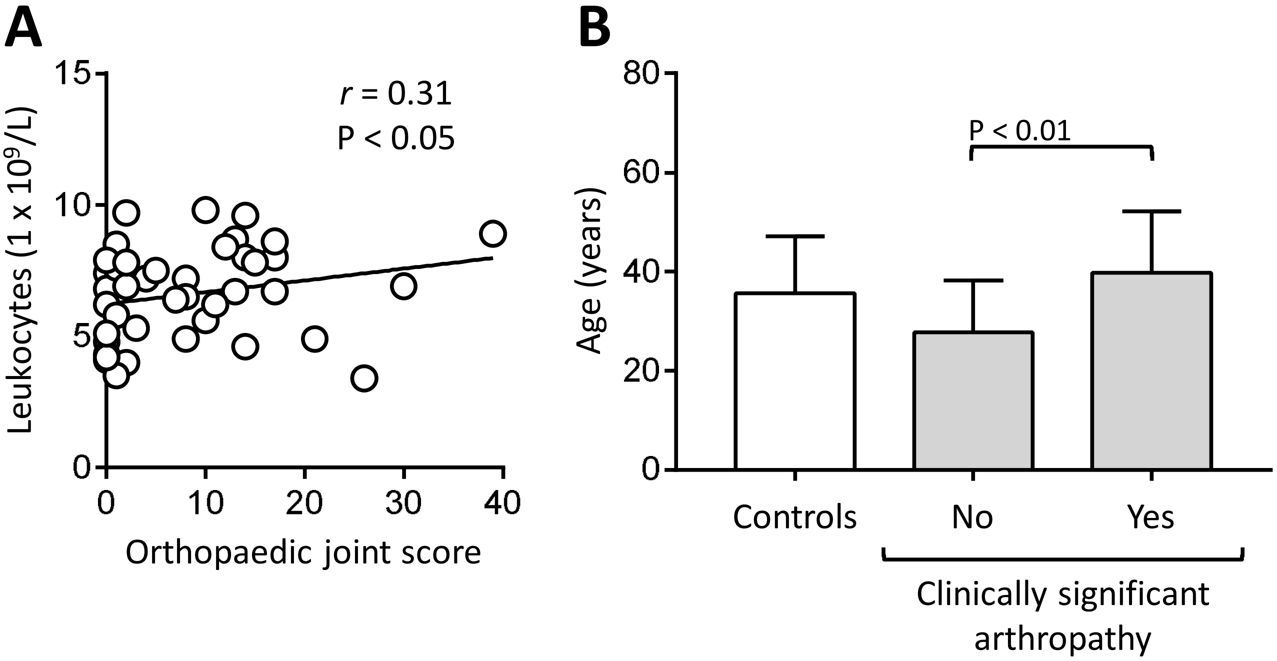

Online Resource 8

	Controls	Patients total cohort	P value*	Patients w/o HBV/HCV	P value*	Patients w/o HBV/HCV, only severe HA/HB	P value*	Patients w/o HBV/HCV, only severe HA	P value*	Patients w/o HBV/HCV, only severe HA, OJS ≤ 4	P value*
**N**	23	43		27		26		22		14	
**Age (years)**	35.6 ± 11.5	33.9 ± 12.8	0.61	28.1 ± 7.0	**< 0.01**	28.0 ± 7.1	**< 0.01**	28.3 ± 7.6	**0.02**	25.1 ± 6.4	**< 0.01**
**LPS TF (MFI)**	4.6 ± 2.6	5.9 ± 3.1	0.08	5.1 ± 2.7	0.53	5.2 ± 2.7	0.46	5.4 ± 2.6	0.32	4.7 ± 2.3	0.93
**LPS TF (%)**	10.9 ± 6.3	14.4 ± 8.8	0.10	12.5 ± 8.3	0.47	12.7 ± 8.3	0.42	13.3 ± 8.4	0.29	11.6 ± 8.1	0.77
**LPS MV TF PCA (AU)**	4.6<br>2.4 – 9.4	10.2<br>4.3 – 17.7	**0.02**	8.4<br>2.5 – 13.3	0.26	8.5<br>2.7 – 13.4	0.18	8.9<br>3.7 – 14.0	0.11	8.4<br>2.2 – 15.5	0.33
**Leukocytes (1 x 10^9/L)**	5.7 ± 1.1	6.6 ± 1.8	**0.03**	6.2 ± 1.7	0.25	6.3 ± 1.7	0.15	6.5 ± 1.6	0.07	6.1 ± 1.5	0.40
**hs-CRP (mg/dL)**	0.05<br>0.04 – 0.10	0.13<br>0.07 – 0.32	**< 0.01**	0.12<br>0.06 – 0.32	**< 0.01**	0.13<br>0.06 – 0.32	**< 0.01**	0.13<br>0.06 – 0.32	**0.01**	0.08<br>0.04 – 0.33	0.20
**IL-6 (ng/L)**	1.0<br>1.0 – 1.8	2.4<br>1.6 – 5.0	**< 0.01**	1.9<br>1.0 – 2.5	**0.04**	1.9<br>1.0 – 3.0	**0.04**	2.1<br>1.0 – 4.5	**0.03**	2.2<br>1.0 – 3.1	0.14
**OJS**	n.d.	5<br>0 – 14	n.a.	1<br>0 – 8	n.a.	2<br>0 – 9	n.a.	1<br>0 – 8	n.a.	0<br>0 – 1	n.a.
